# Thymidine kinase 1 through the ages: a comprehensive review

**DOI:** 10.1186/s13578-020-00493-1

**Published:** 2020-11-27

**Authors:** Eliza E. Bitter, Michelle H. Townsend, Rachel Erickson, Carolyn Allen, Kim L. O’Neill

**Affiliations:** Department of Microbiology and Molecular Biology, Brigham Young University, 701 E University Pkwy, LSB room 4007, Provo, UT 84602 USA

**Keywords:** Thymidine kinase 1, TK1, Biomarker, Regulation, Tumorigenesis, Assay

## Abstract

Proliferation markers, such as proliferating cell nuclear antigen (PCNA), Ki-67, and thymidine kinase 1 (TK1), have potential as diagnostic tools and as prognostic factors in assessing cancer treatment and disease progression. TK1 is involved in cellular proliferation through the recovery of the nucleotide thymidine in the DNA salvage pathway. TK1 upregulation has been found to be an early event in cancer development. In addition, serum levels of TK1 have been shown to be tied to cancer stage, so that higher levels of TK1 indicate a more serious prognosis. As a result of these findings and others, TK1 is not only a potentially viable biomarker for cancer recurrence, treatment monitoring, and survival, but is potentially more advantageous than current biomarkers. Compared to other proliferation markers, TK1 levels during S phase more accurately determine the rate of DNA synthesis in actively dividing tumors. Several reviews of TK1 elaborate on various assays that have been developed to measure levels in the serum of cancer patients in clinical settings. In this review, we include a brief history of important TK1 discoveries and findings, a comprehensive overview of TK1 regulation at DNA to protein levels, and recent findings that indicate TK1’s potential role in cancer pathogenesis and its growing potential as a tumor biomarker and therapeutic target.

## Background

Thymidine kinase 1 (TK1) is a DNA salvage pathway enzyme involved in regenerating thymidine for DNA synthesis and DNA damage. Thymidine is transferred from the extracellular space across the cell membrane by facilitated diffusion and is converted to its monophosphate form (dTMP) within the cytosol by TK1 [1, 2]. Successive enzymes within the cytosol then convert dTMP to its triphosphate form deoxythymidine triphosphate (dTTP) prior to DNA replication. Nucleotides such as dTTP are passively imported into the nucleus through a nuclear pore complex for DNA synthesis and transcription [3]. The de novo pathway is an alternative for regenerating nucleotides but it is anabolic in nature and therefore less favorable when conserving cell energy. During de novo synthesis, deoxyuridine monophosphate (dUMP) is converted to dTMP by thymidylate synthase in the presence of folic acid and vitamin B12 [4]. Because the salvage pathway is less energetically expensive, it is usually the preferred generation pathway within the cell [5]. With the availability of two pathways for dTTP generation, TK1 is not essential for cell viability [6].

Aside from DNA synthesis, TK1 is essential to cell repair following DNA damage. Because TK1 is necessary for the formation of nucleotides outside of the S phase, it is vital to the process whereby pools of dTTP are generated to replace damaged nucleotides for DNA repair [6–8]. For example, TK1 is essential for DNA repair as demonstrated in p53-null colorectal adenoma HCT-116 cells [6]. Cellular DNA damage through radiation or chemotherapeutic agents is followed by significant increases in TK1 levels [7–10], and depletion of TK1 in cells exposed to DNA damage can lead to cell death [6–8].

Regulation of cell cycle factors, including TK1, is crucial for cell homeostasis. Mutations or dysregulation of cell cycle proteins is a major cause of tumorigenesis [11–14]. As early as the 1960s, fetal TK (TK1) activity was shown to be elevated in tumors [15] and TK1 is abnormally high in the sera of several different cancer types including lung, colon, breast, and prostate [7, 16–21]. The increased levels of TK1 are likely caused by a missing C-terminal regulatory region on the translated protein [22–24]. High levels of TK1 protein in cancer sera may potentially be utilized together with other pathological indicators such as biopsies, laboratory tests, and radiological imaging to determine cancer diagnosis and prognosis. Recent findings indicate that, beyond its role as a cancer cell proliferation biomarker, intracellular TK1 is linked to cancer cell invasion and progression [25–27]. The mechanism behind intracellular TK1 upregulation has not been identified nor has its links to cancer progression been fully explored. Overexpression of TK1 may not only be a byproduct of cancer cell processes, but part of selection processes that aid cancer cell progression. TK1-supported tumor growth has been shown in lung adenocarcinoma and breast cancer cell lines; bioinformatical evidence suggests similar TK1 influence in adrenocortical carcinoma and prostate cancer patients [26–28].

Specific pathways and protein interactions of TK1 with other factors that lead to tumor cell progression have not been extensively explored. Fully elucidating the mechanisms behind TK1 elevation in cancer cells and its correlation to cancer progression begins with understanding the regulatory mechanisms of TK1 expression.

## Discovery and characterization of TK1

**Fig. 1:**
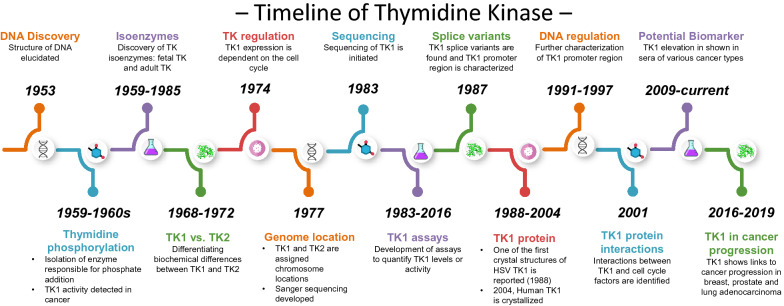
TK1 timeline: a summary of major discoveries regarding the functions, regulation, and characterization of TK1

The discovery of DNA and the subsequent discovery of DNA replication revealed that nucleotide incorporation is preceded by nucleotide phosphorylation (Fig. 1).

During the 1960s, enzymes responsible for the addition of 5′ phosphate to thymidine-deoxyribose were purified and identified as thymidine monophosphate kinase (TMP) or thymidine deoxyribose kinase (TdR kinase) [29–32]. Later research groups found differences between the TK isoenzymes in highly proliferative fetal tissue and adult tissue. They identified two isoenzymes of thymidine kinase that were termed fetal TK (TK1) and adult TK (TK2) [33–36]. Originally, TK1 and TK2 were thought only to be expressed in fetal or adult tissue respectively. TK1 was largely explored in fetal rat liver and found to be present in the highly proliferative tissue, whereas postneonatal tissue had decreased or undetectable TK1 activity while TK2 activity was still detectable [29, 31, 33, 34, 37, 38].

Klemperer and Haynes were among the first to determine biochemical differences between TK1 and TK2 including stability during gel filtration and activity at varying pH [36]. These differences were confirmed later by Taylor et al. [39] in 1972 and expanded to include differences in heat stability, electrophoretic properties, inhibition by deoxycytidine triphosphate, and ability to use nucleotides other than ATP as phosphate donors. During the late 1970s, Elsevier et al. [40] and Willecke et al. [41] assigned chromosome locations for TK1 and TK2. TK1 location was identified on chromosome 17 (17q25.3) and TK2 on chromosome 16 (16q21). In addition to being differentiated by chromosomal location, TK1 and TK2 were further distinguished by cellular location and chemical characteristics. In 1974, Alder et al. and Bello et al. demonstrated that TK1 expression was dependent on cell cycle regulation and that it peaked during S phase within the cytosol [1, 42]. This research provided evidence that TK1 is not solely expressed in highly proliferative tissue, such as in developing fetuses. TK2 was later shown to be responsible for mitochondrial DNA replication [43, 44] and not dependent on cell cycle regulation [43].

Further studies identified additional differences between human TK isoenzymes and herpes simplex virus (HSV) TK [45, 46]. TK thymidine kinase from the HSV was compared to human TK1 and found to be molecularly different; later groups studied substrate specificity and established differences between TK1, TK2, and HSV TK [22, 47]. Technologies employing these molecular differences [48–52] are important discoveries that are continually being studied for application in cancer treatment [53–57].

**Fig. 2:**
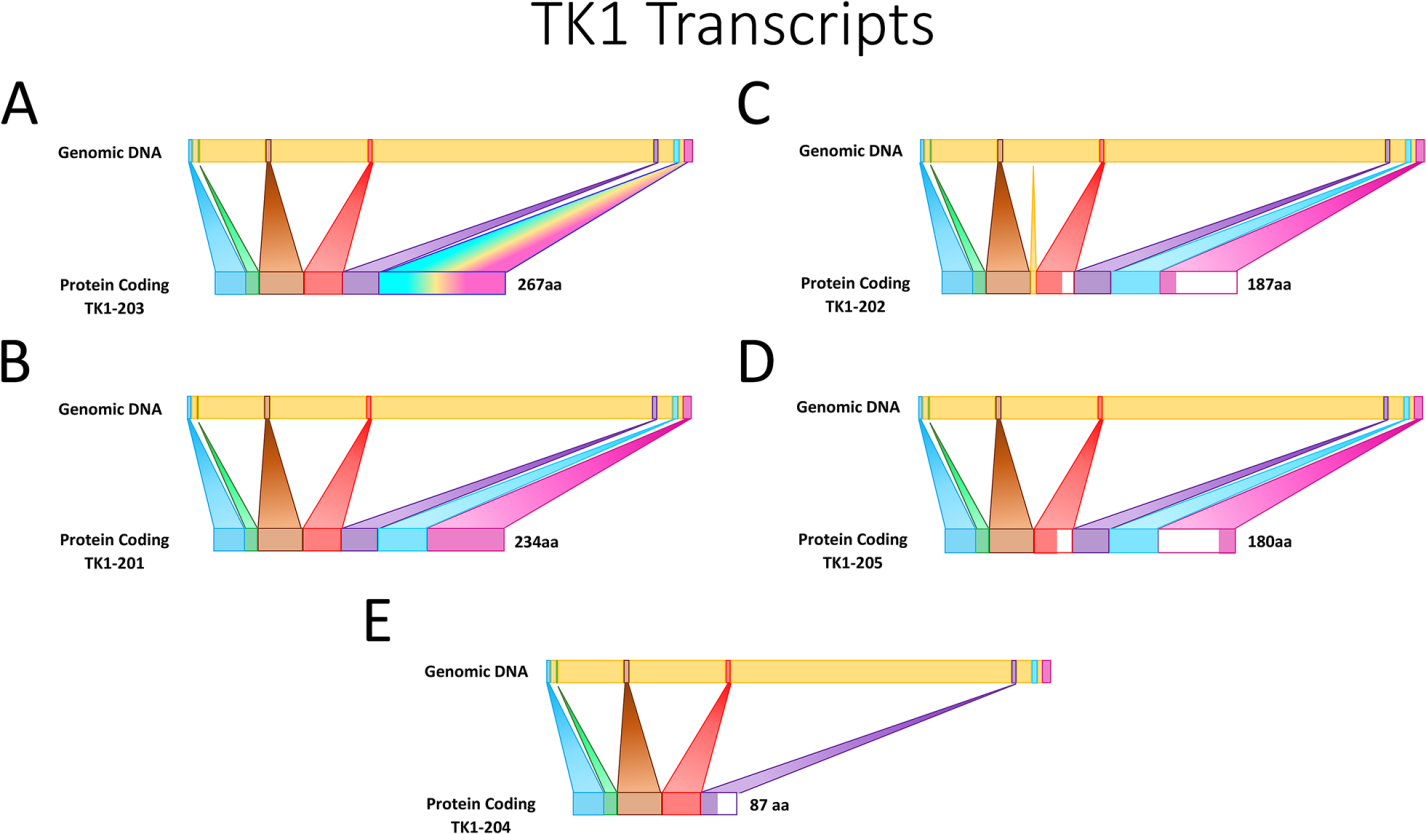
TK1 transcripts: a visual depiction of TK1 transcript variants (**a**–**e**). Protein function is only reported for the active TK1 enzyme which is represented by transcript B

The human TK1 gene is composed of seven exons [58]. There are six splice variants of TK1, five of which are protein-coding (Fig. 2).

Splice variant 1, referred to as NM_003258.5 on NCBI, is 234 amino acids in length [22]. The combination of four chains of splice variant 1 gives rise to the active enzyme and tetramer of TK1 which has a molecular weight of about 100 kDa. TK1 can be present in various forms but is often found as a dimer in a quiescent state and as a tetramer when activated [22]. The other four protein-coding transcripts of TK1 have not been widely explored and no additional roles of the variants have been noted.

In 1988, one of the first crystal structures of TK1 was isolated from the herpes simplex virus by Sanderson [59]. In 2004, Welin et al. [60] successfully crystallized human TK1 complexed with deoxythymidine triphosphate (dTTP), providing further insights into its structure. Each chain or subunit of the tetramer contains two domains: an α/β domain and a small zinc-containing domain [60], meaning that the active enzyme has four active sites. The location of the substrate-binding pocket and active site of TK1 is buried between the α/β and zinc-containing domains [60]. There are three key residues noted to be involved in the active site for thymidine binding, Glu98, Phe128, and Tyr181 [60–63]. The interaction of a phosphate group with thymidine is supported by four cysteine-zinc metal-binding sites at positions Cys153, Cys156, Cys185, and Cys188 [60–63]. Lastly, the binding site of ATP occurs at positions 26–33, 58–60, and 97–100 [60–63] (Fig. 1).

## TK1 regulation at the DNA, mRNA, and protein levels

**Fig. 3:**
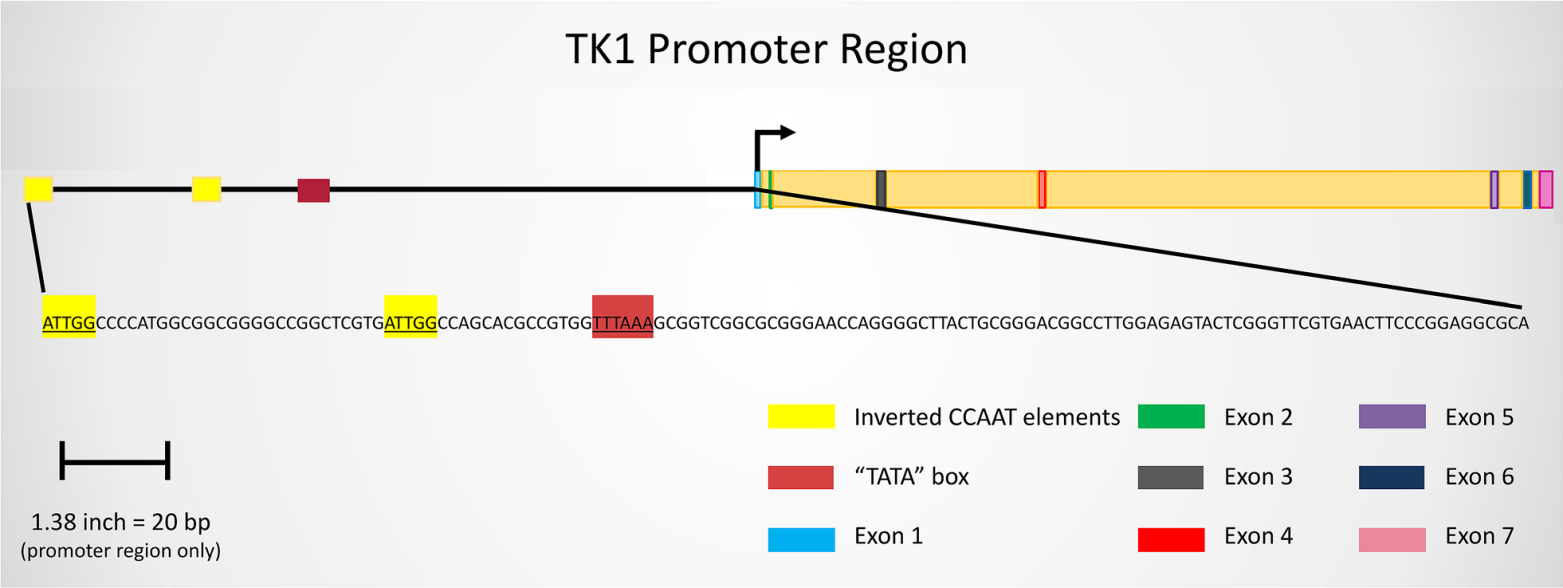
TK1 promoter: a visual depiction of well-documented regulatory regions for the transcription of the TK1 gene

The initial sequencing of the TK1 gene was performed by Bradshaw in 1983, who also identified a 16-kilobase region within the 5′ end region that induced S phase activity of the TK1 gene [64]. This was followed by the identification of specific sequences of regulatory elements within the TK1 coding region. In 1987, Flemington et al. were able to identify the promoter region of TK1 by fusing 5′ regions of the gene to the chloramphenicol acetyltransferase (CAT) gene and assaying for CAT activity in transfected mouse L cells [58]. Transcription signals within the 5′ region contain TATAA, CCAAT, and G-C elements [58, 65]. One of these elements, a 12-base pair inverted CCAAT repeat, has been established as a transcriptional protein binding site [58, 65, 66]. The inverted CCAAT motif responsible for transcriptional activation of TK1 is most likely recognized by the transcription factor CCAAT-binding protein CP1 due to sequence homology with other genes regulated by CP1 [4, 65]. Work performed by Bradshaw and Flemington et al. was followed by studies performed by Kim and Lee [58, 64, 65], who identified a 70 bp region in the promoter of the human TK1 gene that influenced cell cycle regulation of TK1 [22, 65] (Fig. 3).

The cell cycle has a plethora of components that manage checkpoints and passage from phase to phase [12, 14, 67–73]. Briefly, there are three checkpoints that a cell progresses through to enter mitosis: G1 (restriction point), G2, and S. Progression through these checkpoints is reliant on cyclins and cyclin-dependent kinases (CDKs) which function as positive or negative regulators. Negative regulators include proteins such as p53, retinoblastoma protein (Rb), and kinase inhibitor p21 [67–69, 74]. It is well established that many cell cycle factors involved in G1 to S phase transition are regulated specifically by the tumor suppressor genes: Rb, p21, and p53 [73, 75–78]. In normal cells, the active hypophosphorylated form of Rb interacts with E2F to prevent transcription of cell proliferation genes such as cyclins, CDKs, and DNA repair proteins [75]. Phosphorylation of Rb leads to the disassociation of Rb and E2F complex and is managed by CDK/cyclin complexes such as CDK4/6-cyclin D and CDK2/cyclin E [79]. This pathway is often referred to as the Rb-E2F pathway. Interactions of TK1 with regulatory factors in this pathway, such as p21, have been demonstrated [80]. Further, TK1 mRNA levels during normal cell function or during the repair of DNA damage have been determined to be regulated by the pRB/E2F pathway [1, 5, 81].

The MAP kinase and p53-p21-DREAM-E2F/CHR pathways have also been shown to affect TK1 expression. Using trametinib to block the MAP kinase pathway in lung adenocarcinoma (LUAD) cells A549, H1299, and H460, Malvi et al. [28] demonstrated that levels of TK1 measured through quantitative reverse transcriptase-PCR were lower in treated cells. Recently, it has been demonstrated that, directly or indirectly at the transcription or protein level, the p53-p21-DREAM (DP, RB-like, E2F4, and MuvB)-E2F/CHR pathway controls over 250 cell cycle genes, including TK1 [9, 10].

P53 within the “DREAM pathway” elicits common p53 functions. The key functions of p53 include regulation of the cell cycle and apoptosis by controlling activation or downregulation of target genes. Cyclin-dependent kinase inhibitor p21 is activated by p53 and halts the cell cycle. Once activated, p21 indirectly influences transcriptional regulation and binds to TK1 [80]. Engeland and Fischer showed p21 leads to hypophosphorylation of Rb and causes the DREAM complex to form; the complex in turn acts as a transcriptional repressor. Genes repressed by this pathway are proposed to be present anywhere from G1 to S phase [9]. The DREAM complex has been shown to bind at promoter cell cycle homology regions (CHR) and E2F transcriptional sites, such as those present in TK1 [9]. The E2F binding site present in the promoter region of TK1 is proposed to be a p53-DREAM target for repression, although it remains unclear how often this pathway is utilized by the cell [9, 10].

Post-translationally, protein levels of TK1 are stabilized by a 40-amino-acid sequence in the carboxyl terminus during S phase; this region also signals degradation of the transcripts during G2 and mitosis [9, 24]. The degradation of the enzyme is mediated by ubiquitin–proteasome-related mechanisms [6, 82]. Specifically, a KEN box located at positions 203–205 in the C-terminal region of human TK1 is targeted by Anaphase-Promoting Complex/Cyclosome-Cdh1 complex, marking it for ubiquitin proteolysis [82]. Cancer mutations or deletions in the C-terminal region abolish cell cycle-specific degradation and allow TK1 to be expressed even in G0 [22–24]. Aside from abolishing cell cycle-specific regulation, Kauffman et al. showed that the enzymatic activity of the truncated human TK1 protein remained unaltered [24]. Zhu et al. analyzed varying lengths of TK1 C-terminal deletions—20 (CΔ20), 40 (CΔ40), and 44 (CΔ44) amino acids—and their effects on TK1 in vitro stability, oligomerization, and enzyme kinetics [23]. Interestingly, they discovered that the deletion of the C-terminal fold increased protein stability and catalytic activity [23]. Protein stability was determined by half-life and measured in minutes and increased in both CΔ40 (335 ± 24) and CΔ44 (500 ± 70) when compared to wild type samples (83 ± 7) [23]. Enzyme kinetics measured per second increased in CΔ40 (9.5 ± 1.19) when compared to wild type (6.3 ± 0.9) [23].

The activity of the full TK1 tetramer is commonly controlled allosterically by levels of dTTP [1, 22, 83, 84]. When dTTP binds, the binding pocket of TK1 is hidden from ATP and thymidine, thereby preventing phosphorylation of thymidine [2, 61, 85, 86]. The optimal activity of TK1 occurs at a pH of about 7.9 [30]. Post-translational modifications such as phosphorylation of serine residues result in activation of TK1 [22–24]. In addition, TK1 activity is seen when high levels of the active enzyme are shown to overcome the inhibitory binding of p21 [80].

## TK1 elevation associated to inflammation and infection

Deficiencies in TK2 are inherited as autosomal recessive mutations and can cause a spectrum of diseases manifesting themselves from infancy to adulthood, but usually as myopathy [87]. No known deficiencies of TK1 have been reported, as defects in TK1 could lead to fetal death in utero. Despite no known diseases caused by TK1 deficiency, levels of the enzyme in serum are connected to infection and inflammation.

In 1984, Gronowitz et al. [88] evaluated serum TK1 activity levels, through the conversion of 1^25^I-iododeoxyuridine (IdUrd), in a variety of human diseases. TK1 levels were found to be 10–40 times higher than normal in patient sera during the acute stage of infectious mononucleosis. In other viral infections, TK1 serum levels were in the normal range except in patients with measles, rubella, varicella, herpes simplex virus, and cytomegalovirus infections.

Infection and inflammation marked by increased TK1 levels have also been shown in canine subjects [89, 90]. Protein sequence homology is high between human and canine TK1; canine models can accordingly be related to human subjects [91]. In one study conducted by Sharif et al. [89] TK1 levels were measured in female canines diagnosed with pyometra (n = 54), a common uterine infection, and healthy subjects (n = 40). TK1 levels were measured and compared with other hematological and biochemical parameters, such as acute phase proteins and inflammatory mediators (C-reactive protein and Prostaglandin F_2α_). TK1 activity levels were higher among dogs with pyometra compared to healthy females. Interestingly, no significant correlations were found between TK1 activity and other serum biomarkers of inflammation (p > 0.05).

In a separate study, Selting et al. [90] showed that the concentration of C-reactive protein and TK1 both increased in dogs with solid and hematological tumors (n = 253) compared to the control (n = 156). In addition, they found that increased levels of TK1 and C-reactive protein also correlated with increasing grades of tumors, and both of these markers when used to evaluate tumor presence and grade correlated with the standard Neoplastic Index. Their results indicate a relationship between TK1 levels and serum biomarkers of inflammation.

## TK1 as a tumor biomarker

TK1 activity was shown to be elevated in tumors as early as the 1960s [15]. Since then, various studies have shown that increased levels of TK1 are found in the sera of many different cancer types including some of the most commonly diagnosed: lung, colon, breast, and prostate [7, 16–21, 92].

In one study, TK1 upregulation was indicated as an early event by Alegre et al. [18] in breast cancer tissue. TK1 levels were measured in breast carcinoma patient tissue samples including simple carcinoma, infiltrating ductal carcinoma, medullary carcinoma, and sclerosing carcinoma. All samples for infiltrating lobular carcinoma (n = 3) and scirrhous carcinoma tissue (n = 11) stained positive for TK1. For simple (n = 30), infiltrating duct (n = 41), and medullary (n = 12) carcinoma tissue samples, 83%, 63%, and 66% respectively stained positive for TK1.

Interestingly, four precancerous breast tissue samples were also analyzed for TK1 levels [18]. This progressive precancerous breast tissue array included normal breast, breast adenosis, sclerosing adenosis, and atypical hyperplasia. Findings of this study showed that 22% of the adenosis samples were positive for TK1, with the earliest indications of increased TK1 levels in sclerosing adenosis—results signifying that TK1 levels could be an early indicator of tumor development. Further comparisons between TK1 levels among all positive samples showed that levels of TK1 progressively increased with cancer stage, indicating a positive correlation between TK1 and cancer stage. In subsequent studies conducted by Alegre et al. [92] it was demonstrated that TK1 was a “universal” marker for malignancy among multiple cancer types involving lung, colon, prostate, esophagus, stomach, liver, and kidney tissues.

Subsequent studies support the potential of utilizing TK1 clinically to determine treatment effectiveness, cancer stage, and prognoses in a wide variety of solid cancer types as well as for AML and ALL patients [92–97]. A clinical study conducted by Nisman et al. in patient samples determined that increased levels of serum TK1 after first and second rounds of chemotherapy in non-small-cell lung cancer (NSCLC) and small-cell lung cancer (SCLC) was indicative of treatment failure and poor overall survival [96]. Huang et al. and Wang et al. also contributed to this theory when they showed that higher rates of occurrence and poorer overall survival of patients with locally advance breast cancer patients and ovarian serous adenocarcinoma patients who had abnormal levels of serum TK1 [98, 99].

In 2011, von Euler and Eriksson published a review paper speaking not only to TK1 structure but its clinical application in solid tumors and hematological malignancies [91]. One hematological study evaluated results from chronic lymphocytic leukemia (CLL) patients (n = 188) in which serum TK1 levels were used to monitor patients treated with fludarabine alone or in combination with prednisone [100]. Measuring serum TK1 activity levels using the TK-REA assay (see Table 1 and “TK1 quantification methods in patient sera”), the researchers discovered that only 45% of patients responded to combination treatment when TK1 levels were ≥ 10 U/L. In comparison, subjects with low TK1 serum levels (< 10 U/L) showed an 83% response. The conclusion drawn from this study proposed using TK1 levels as a reference when planning CLL patient treatment, and specifically when considering more aggressive treatment options for patients with TK1 levels ≥ 10 U/L.

**Table 1:** Summary of TK1 assays and reported results

TK1 assay	Brief description	Measurement units	Normal versus cancer
[^3^H] Deoxythymidine phosphorylation	Samples are incubated with ATP, DTT, MgCl_2_ and [^3^H]-labeled deoxythymidine. Labeled thymidine converted by TK1 to [^3^H]-dTMP is quantified to determine TK1 levels based on standard curves	pmol/min/mL	Normal: 0.6–3.1 pmol/min/mL Cancer: 0.9–48 pmol/min/mL [127]
TK-radio enzymatic assay (TK-REA)	Assay utilizes [^125^I]-IUdR, an analog substrate of TK1, to measure TK1 activity. The output of this assay gives linear turnover of substrate for cellular dTK-F (TK1). 1 unit enzyme = 1.2 × 10–18 kat	U/L	*Normal: 2.5 × 10^–1^ U/L *Low-grade malignancy: 4.6 U/L *Intermediate grade: 28.8 U/L *High grade: 45 U/L [88]
TK1-Liaison	3-Azido-3-deoxythymidine (AZT) is converted to AZTMP by TK1. Phosphorylation is quantified through a competitive ELISA using anti-AZTMP antibody and AZTMP-labeled peroxidase. Color development is inversely proportional to TK1 activity and reported as U/L	U/L	Normal: 2.4–5.6 U/L Cancer: 4.0–8.2 U/L [114]
DiviTum™	BrdU is converted to BrdUMP by TK1 and then to BrdUTP by yeast enzymes. The dUTP formed during the reaction is washed over immobilized polyA strands. BrdUTP is detected with an anti-BrdU-antibody conjugated to alkaline phosphatase. The color development is measured at 405 nm	Divitum units (Du)/L	Normal: 9–33 Du/L Cancer: 20–92 Du/L [114]
Chemiluminescent dot blot (IgY)	A nitrocellulose membrane is blotted with ~ 3 µL of sample and dilutions of a TK1 standard peptide. The membrane is probed with anti-TK1 chicken antibody (IgY). Blot intensities are measured using a charge-coupled device camera. A standard curve is used to infer sample measurements	pM	Normal: < 2.0 pM [124]
TK1-210	Antibodies used in this sandwich ELISA are designed against the exposed C-terminal end of TK1. Monoclonal antibodies against TK1 are immobilized on a microtiter plate and used to capture TK1 peptides. Secondary antibody conjugated to biotin is used to detect TK1. Strept-HRP is used to develop color (absorbance 450 nm)	ng/mL	Normal: 0.17–0.33 ng/mL Cancer: 0.17–9.9 ng/mL [127]

^*^Presented as average value

A comment published by McCarney and Malorni presented early results from a study in which TK1 activity levels were used to identify patient response to CDK4/6 inhibitors [101]. Previous research by Bonechi et al. and McCartney et al. had shown that TK1 can be both a prognostic and a resistance marker for HR-positive, HER2 negative breast cancer patients receiving endocrine therapy [94, 102]. CDK 4/6 inhibitor therapy has been successful in promoting progression-free survival (PFS) in HR-positive, HER2-negative metastatic breast cancer both initially and in later settings following endocrine therapy. Patient plasma was gathered from a Phase 2 clinical trial (TREnd, NCT02549430) and analyzed for TK1 activity. Enrolled patients were treated with a combination of endocrine therapy and palbociclib (CDK 4/6 inhibitor) or with palbociclib only. Early data showed that patients with an increased TK1 activity following one cycle of palbociclib had a significantly worse PFS when compared to patients with stable or decreased activity levels (p = 0.002). To date, unfortunately, there are no predictive or prognostic biomarkers that can be used for monitoring or predicting patient response to CDK 4/6 inhibitors. In future research, TK1 activity will be assessed at baseline and compared to the clinical outcome in PYTHIA (NCT02536742), a Phase 2 clinical trial and single-arm study evaluating HR-positive, HER2-negative metastatic disease when treated with palbociclib plus fulvestrant.

When evaluating TK1 as a tumor biomarker in comparison with other validated markers, researchers have found that TK1 possesses significant advantages in comparison to such biomarkers as Ki-67. Most notably, perhaps, cancer patient serum is readily accessible to clinicians who can easily test for TK1 levels as an indicator of disease severity [7]. Additionally, Ki-67 is present during all phases of the cell cycle, peaking during G2, and PCNA is mostly present during later G1 [17, 103, 104]. In comparison, TK1 is more informative in that it closely mimics the rate of DNA synthesis due to its peak expression during S phase [105]. To summarize, other tissue proliferation biomarkers such as Ki-67 have been shown to be inconsistent in certain cancer types, whereas TK1 being closely linked to cell growth stage more closely reflects tumor proliferation [91, 99].

Careful analysis of prostate tumors, ovarian serous adenocarcinoma, and lung adenocarcinoma have demonstrated that TK1 is also more sensitive and reliable as a biomarker than Ki-67 and PSA [97, 99, 105]. For example, Li et al. found when studying the prognosis of men with prostate cancer that serum TK1 levels correlated with Gleason scores whereas PSA levels did not [97]. Furthermore, tissue staining for TK1 has been used to identify activated tumor vessels involved in angiogenesis in colorectal cancer (CRC) patients; indeed, in studies involving double immunostaining for both CD31 and TK1, results showed TK1 to be a highly specific marker for activated vessels [106]. Tian et al. [107] demonstrated the diagnostic and prognostic capabilities of TK1 concentrations over those of other biomarkers in malignant pleural effusion (MPE), especially when compared to parallel biomarker readings of benign pleural effusion (BPE). Within their study, Tian et al. compared pleural TK1 to the measurement of serum TK1 levels in 210 patients whose samples were classified as MPE or BPE. While this study was limited to hospitalized patients, results demonstrated that TK1 had the highest diagnostic accuracy compared to such other markers as neuron‐specific enolase (NSE), CEA, and cytokeratin fragment 19 (CYFRA 21-1). Again, the measurement of TK1 through a pleural effusion procedure is less invasive than other methods for MPE diagnosis.

Despite its advantages as a biomarker, TK1 is perhaps best used in tandem with other markers or procedures, given that elevated levels of TK1 in the serum may be caused by inflammation or infection and not only by cancer [7]. If elevated levels of TK1 are found in the sera of patients, further clinical investigation is required to determine if the elevation is a result of cancer or other cause. In their meta-analysis, Xiang et al. [21] concluded that TK1 as a single diagnostic proliferating tumor biomarker was not always a significant indicator of disease, thus recommending its diagnostic use in combination with other tumor markers.

Given that elevated tissue expression of TK1 has been shown to be present in multiple cancer types and an early event in cancer development, and given that such expression levels have been shown to be associated with tumor grade, TK1 measurements in serum could be developed as a valuable prognostic tool when used with other pathological tests. Several studies have shown its effectiveness as a tumor biomarker and its advantages in comparison to other biomarkers. As a result of such findings, serum TK1 levels have obvious potential as a biomarker for cancer disease management.

## TK1 quantification methods in patient sera

The presence of elevated levels of TK1 in cancer sera has led to the development of assays to quantify TK1 levels in clinical settings (Table 1). Current reviews evaluating past and current TK1 assays include those written by Jagarlamudi et al. and Zhou et al. [7, 17].

Prior to more efficient methods, TK1 activity assays were based on anion exchange chromatography to separate phosphorylated nucleoside products from unphosphorylated nucleoside substrate in TK1 reactions [108–110]. These methods were limited in the number of samples that could be processed and involved large amounts of solvents and special handling [110].

The [^3^H]-deoxythymidine phosphorylation assay was one of the earliest methods used to measure TK1 activity through radioactivity, thereby avoiding excessive use of reagents and increasing the number of samples available for processing [110]. For several years, this test was considered to be the “gold standard” for determining TK1 activity [111]. In the test, samples are incubated with ATP, DTT, MgCl_2_, and [^3^H]-labeled deoxythymidine. Labeled thymidine converted by TK1 to [^3^H]-dTMP is separated using a precipitate, leaving unphosphorylated product in solution. The precipitate is subsequently quantified to determine TK1 activity levels. Wolcott et al. showed the specificity and sensitivity of the assay using mouse L cell TK1 negative cell line (LMTK^−^) and TK1 positive cell line (LHTK^+^). The LHTK^+^ cells were derived from LMTK^−^ cells transfected with the HSV-1 TK gene [110]. When HSV-1 TK substrates [^125^I]-iododeoxyuridine, [^125^I]-iododeoxycytidine, and [^3^H] thymidine were added to LMTK^−^ and LHTK^+^ cytosol samples, the lack of detectable TK1 activity in LMTK^−^ cells showed the specificity of the assay. In a separate experiment, TK1 activity was determined in serially diluted cytosols of both LMTK^−^ and LHTK^+^ cells. The TK1 activity was shown to increase linearly with increasing protein concentration, and as little as 0.1 µg of total cytosolic TK1 protein was detected [110].

Another early assay for TK activity was also a radiometric enzyme activity assay. This assay employed the use of the thymidine analog [^125^I]-5-iodo-2-deoxyuridine (IUdR) as a substrate and was named the TK-radio enzymatic assay (TK-REA) [112]. The level of phosphorylated IUdR was quantified in enzyme activity units (U) which provided a linear curve of enzyme activity that could be used to determine cellular and serum TK. Gronowitz et al. [112] indicated the assay’s ability to determine survival, prognosis, and treatment efficacy in blood cancers such as leukemia and Hodgkin’s and non-Hodgkin’s Lymphoma.

The radioactive tracers needed for assays such as [^3^H]-deoxythymidine phosphorylation or TK-REA made them untranslatable for clinical use. Subsequent groups relied on the substrate specificity of TK1 and utilized non-radioactive thymidine analogs to radioactive tracers. Two of these assays are the TK-Liaison assay and the DiviTum™ assay.

The analog used in the TK-Liaison assay (Diasorin^®^) is 3-azido-3-deoxythymidine (AZT) which is converted to AZTMP through the TK1 present in samples. The phosphorylation event is quantified through a competitive ELISA using anti-AZTMP antibody and AZTMP-labeled peroxidase. The color development is inversely proportional to the TK1 activity [113]. Levels of TK1 enzyme activity are determined by a calibration standard against AZTMP and reported in units of U/L. Having an easily built standard based on AZTMP levels is one of the advantages of the TK-Liaison assay. The authors suggest that there may be advantage in TK activity assays over antibody-based methods [113]. One general concern is antibody sensitivity when measuring TK1 in healthy individuals who may have low concentrations of serum TK1. In addition, antibodies may react with both active and inactive or degraded TK1 that may be present [113]. Distinguishing between active, inactive, or degraded forms of TK1 in serum may be important for accurate diagnosis.

Ohrvik et al. [113] studied the clinical correlation of the TK-Liaison and TK-REA assays on sera from patients with hematologic malignancies including non-Hodgkin lymphoma (n = 54), Hodgkin lymphoma (n = 17), follicular non-Hodgkin lymphoma (n = 2), chronic lymphocytic leukemia (n = 4), and multiple myeloma (n = 1). From their study, they determined there was a high correlation between the two methods in determining TK1 levels; they also found that results from the TK-Liaison were reproducible and that the Liaison method did not involve radioactivity. In addition, they showed that the cross-determination with TK2 was low (< 0.1%) compared to the TK-REA assay (< 4%) [113].

The DiviTum™ assay developed by Biovica^®^ is another nonradiometric assay. It measures bromodeoxyuridine or 5-bromo-2′-deoxyuridine (BrdU) incorporation into DNA through an anti-BrdUTP antibody [114]. Firstly, BrdU is phosphorylated to its monophosphate form (BrdUMP) by TK present in samples. BrdUMP is then phosphorylated to its triphosphate form (BrdUTP) by yeast kinases that are present in the reaction solution. The BrdUTP is then captured by PolyA strands covalently immobilized to the well of a microtiter plate. Bound BrdUTP is detected with an anti-BrdUTP monoclonal antibody conjugated to alkaline phosphatase (AP). The amount of AP is evaluated using a chromogenic substrate. The color developed is proportional to the TK activity present in the samples and is measured at 405 nm. The TK activity is determined from a calibration curve established using TK1 standards, the units are expressed as DiviTum units/L (Du/L). The TK activity measured in the DiviTum assay is total TK.

Bonechi et al. [102] used the DiviTum assay to study the response of patients with metastatic breast cancer (MBC) to endocrine therapy (ET). Overall, their results indicated the capability of the DiviTum assay in a clinical setting. Samples used in their study included cell lysate of three HR^+^/HER2 negative breast cancer cell lines treated with endocrine therapy (ET) (MCF7, T47D, and ZR-75-1) and plasma from HR^+^/HER2 negative patients receiving ET (n = 31). Plasma samples were collected at ET initiation, at 4 weeks following ET, and at the time of progressing disease. High TK1 levels significantly correlated with increased circulating tumor cell (CTC) counts and low levels indicated improved progression-free survival state [102]. Their results showed the potential of TK1 as a prognostic, predictive, and monitoring marker of early ET response in HR^+^/HER2 negative MBC patients [102].

Sera from patients with metastatic breast cancer were measured for total TK activity using the DiviTum assay at baseline (n = 142), 1 month (n = 134), 3 months (n = 122), and 6 months (n = 104) [115]. Overall, median serum TK activity (sTK) levels were reduced with systemic treatment. When evaluating sTK levels specific to treatment, patients were divided into three groups: chemotherapy (ChT), endocrine therapy (ET), and HER2 directed therapy (in combination with ChT or ET). There were varying responses based on treatment type. Patients receiving ChT had an increased sTK median at 1 month of treatment (874 Du/L) when compared to baseline (420 Du/L) (p < 0.001); at 3 months the levels dropped back down (759 Du/L) and at 6 months the median level fell below baseline (387 Du/L). Those receiving ET had significantly reduced TK levels at 1 month (p = 0.008) and progressively reduced at 3 and 6 months when compared to baseline. HER2 directed therapy showed the most drastic changes in median TK levels when compared to baseline (1037 Du/L). At 1 month the median level dropped to 913 Du/L, at 3 months to 197 Du/L, and at 6 months to 107 Du/L. Measured TK levels were compared against other clinicopathological factors such as circulating tumor cells (CTCs), the number of metastatic loci, and therapy type. High levels correlated to high counts of CTCs and increased metastatic loci (≥ 3). High TK levels at baseline were also associated with worse PFS (p < 0.001).

Subsequent groups developed antibody-based methods such as chemiluminescent dot blots and ELISAs to measure TK1 protein rather than relying on TK1 activity [116–118]. The ability to directly measure TK1 protein decreased processing times to determine prognosis, treatment monitoring, follow-up and survival, and relapse [7, 116].

In 2000 and 2003, two different groups developed assays using antibodies against TK1 for an enhanced chemiluminescent (ECL) dot blot detection system [116, 118]. Of these, one group used egg yolk immunoglobulins (IgY) against a 31-amino acid peptide in the C-terminal region of TK1 [116] and the other used a mouse polyclonal antibody generated against a 15-amino acid peptide of the C-terminal region of TK1 [118]. Wu et al., which utilized the IgY antibodies, showed that the lower limit of detection can detect as little as 0.1 picograms of TK1 in 3 μL sera. In both ECL assays, samples are applied to a nitrocellulose membrane (1–5 µL). In addition, TK1 standards—a recombinant TK1 [118] or a TK1 peptide [116]—are applied onto a nitrocellulose membrane in varying concentrations. The membrane is then blocked in a buffered solution of non-fat milk and probed with primary antibody. A secondary biotinylated antibody is then added (anti-mouse or anti-chicken) and developed using an HRP streptavidin substrate. Spot intensities are then determined using a laser densitometer [118] or a charge-coupled device camera [116] depending on the ECL assay. Finally, standard curves generated from the TK1 standard measurements are used to extrapolate sample concentrations.

The ECL assay using the IgY antibodies has been studied the most widely in clinical application. Originally, the IgY antibodies were used to test TK1 levels in sera of patients with gastric cancer (GC). When comparing preoperative patients (n = 31) with healthy controls (n = 62), TK1 levels were significantly higher in GC patients (p < 0.01) [116]. Subsequently, several research teams evaluated the efficacy of this assay in patients starting from 2005—when combined, over 150,000 patients were evaluated [119–122]. In 2016, Cao et al. [119] published their findings from a study involving 14,960 patients. They showed using the ECL dot blot system that the mean pretreatment standard score (z-score) comparing the standard deviations of the raw scores to the mean for serum TK1 levels between men and women was 0.01 ± 0.99 [119]. A retrospective study in 2016 conducted by Wang et al. [122] showed the value of the ECL when they examined serum TK1 (STK1) and compared it to carcinoembryonic antigen (CEA) and alpha-fetoprotein (AFP) in cancer screening in a cohort of 56,286 patients in Quanzhou, China. From their analysis, they concluded that STK1 was more sensitive for detecting malignancies than CEA and AFP and had potential as a prognostic biomarker associated with an increased risk of death while CEA and AFP did not [122]. In a follow-up study performed by Wang et al. [123] they evaluated 56,178 patients to test if STK1 levels could detect pre-malignant disease. Using a cut-off value of 2 pM they discovered that elevated STK1 levels were associated with premalignant disease for liver and prostate cancer patients.

Chen et al. [124] used the IgY ECL assay for sTK1 measurement in a study on 35,365 serum samples from a health screen and found that 2 pM was a reasonable risk threshold to determine the risk of developing a malignant tumor later in life. Those with sTK1 levels above 2 pM had a 3 to 5 times greater risk of developing new malignancies within 11 years.

A meta-analysis performed by Lou et al. [120] evaluated twenty studies in which the half-life of serum TK1 concentration (STK1c), measured using the IgY ECL assay, was used to monitor the response of lung cancer patients to extensive surgery. In their analysis, they showed that STK1c was reduced in patients following extensive surgery by 41.7% (p < 0.00001) and that STK1 levels were significantly higher in patients with lung cancer when compared to healthy persons or those with benign disease (p < 0.00001). They concluded that the half-life of STK1c is a useful parameter for evaluating surgical response in lung cancer patients and that STK1c may be beneficial for early detection of disease. A separate meta-analysis performed by Dang et al. studied whether serum TK1 protein levels (STK1p) measured using the ECL assay could distinguish between healthy patients (n = 1,701), benign colorectal tumors (n = 774), and colorectal cancer (CRC) (n = 1836) [125]. Twenty studies spanning from 2009 to 2019 were collected and evaluated. Their results revealed that STK1p levels were effective in distinguishing between all three patient groups (p < 0.00001) and that STK1p levels decreased by 40% following surgery (p < 0.00001).

Despite extensive clinical studies involving the IgY ECL test, together with the ease of its administration and its relatively short processing time, the test may be unreliable in certain cases in quantifying levels of TK1. He et al. [118] studied the assay’s capability to distinguish between breast cancer patients that included preoperative (n = 17), postoperative tumor-free (n = 38), metastases to lymph nodes (n = 10, N1–2), benign tumors (n = 21), and healthy volunteers (n = 11). While there were significant differences in measured results among various groups, there were also overlaps between results from specific groups, such as between preoperative and healthy patients, making them difficult to distinguish [118].

An alternative immunoassay system to improve the specificity and sensitivity for the measurement of serum TK1 levels involves ELISA development. The first study using a commercial TK1 sandwich ELISA was published in 2009 by Carlsson et al. [126]. Since this ELISA exhibited relatively low specificity and sensitivity for serum TK, a new version was developed by the AroCell^®^ company and is known as the AroCell TK-210 ELISA (Table 1) [127].

Antibodies utilized in this assay were created against the 210-amino acid epitope on the exposed C-terminal end of TK1 (amino acid sequence 194–225) [17, 128]. A problem when measuring TK1 in serum is the presence of TK1 complexes which prevent accurate detection of TK1. The TK-210 ELISA solves this issue by first pre-incubating serum with a dilution buffer which breaks down these complexes and allows for the TK1 to be accessible for immunoassay. In the assay, a monoclonal antibody is used to capture the TK1 peptides in the serum sample. A second TK1 antibody conjugated to biotin is added, then a streptavidin-horse-radish peroxidase (streptavidin-HRP) conjugate is added and then a TMP substrate. The stronger the color development, the higher the concentration of TK1 in the sample. The color development is analyzed at 450 nm. TK1 concentrations are read from a calibration curve generated with TK1 standards and reported as µg/L. In a study performed by Kumar et al. [127] the TK1-210 assay was better able to distinguish healthy from breast cancer patients when compared to an enzyme activity assay. Overall, a major advantage of the assay is an improved ability to more accurately measure TK1 levels associated with solid tumors [129].

Between 2008 and 2011, 150 women with primary locally advanced HER2-negative breast cancer were enrolled in a study evaluating pathological tumor response to breast cancer treatment [130]. The response was monitored in localized breast cancer patients (n = 104) by measurement of cell-loss, which was defined as the ratio of serum concentration of TK1 (ng × mL^−1^) and tumor volume. TK1 levels were measured using the AroCell TK210 ELISA. Identifying early pathological complete response (pCR) to neoadjuvant chemotherapy (NACT) indicates a more favorable prognosis and could aid clinicians in more effective treatment plans. In addition, NACT reduces the extent of surgery for patients which allows for higher breast conservation. Results from this study showed that TK1 could be used as an early metric for cell-loss and tumor volume in localized breast cancer patients: of the 104 patients monitored 24 achieved pCR.

Immunoassay kits as those mentioned above create opportunities for clinical use to measure TK1. Unfortunately, measuring TK1 clinically is still not routine practice. Present TK1 assay kits continue to require lengthy processing times and do not accurately measure all TK1 forms.

A study performed by Velazquez et al. [131] evaluated monoclonal antibodies designed against one of six different epitopes in the tetrameric form of human TK1. Most existing antibodies targeting TK1 are against the cell cycle regulatory C-terminus which may limit TK1 detection to only certain forms of the enzyme. These antibodies were validated through Western blot, siRNA TK1 knockdown, ELISA, and flow cytometry testing in lung, breast, colon, and prostate cancer cells. From their analysis, Velazquez et al. deduced that antibodies against epitopes two, five, and six showed the highest affinity to TK1 and could potentially detect multiple forms of TK1. Aside from recommending use of these antibodies in a cancer screening format, the study indicated that TK1 may have potential as an immunotherapeutic target for antibody-based and adoptive cell therapies [131].

The increasing knowledge on TK1 as a tumor biomarker and improved measurement of TK1 levels in patient sera have created opportunities for possible clinical adoption of TK1 in patient monitoring. As noted above, several studies that have utilized different methods for TK1 measurement have shown that serum TK1 levels are valuable for making prognoses and patient monitoring.

## Recent discoveries about TK1

A review by Topolcan and Holubec [16] explained that intracellular TK1 has and could be targeted in cancer treatment and that TK1 may influence cancer development. Identified roles of TK1 in DNA synthesis and repair have been well characterized. “However, even with abundant evidence for the overexpression of TK1 (protein) in a wide variety of cancer and the association of this protein with poor prognosis, no study thus far has analyzed the functional implication of TK1 inhibition on tumor growth and progression” [28]. To date, there is no identified functional difference between TK1 protein and TK1 mRNA in normal and tumor cells. More recent in vitro and bioinformatic studies aim to identify additional pathways where TK1 could influence cancer pathogenesis.

Malvi et al. [28] determined through the shRNA knockdown of TK1 in lung adenocarcinoma (LUAD) cell lines (A549, H1299, and H460) that TK1 can promote LUAD tumor growth and metastasis through activating Rho GTPase and growth and differentiation factor 15 (GDF15). Through Illumina BeadChip array, gene expression data from TK1 knockdown cells revealed that five genes––growth and differentiation factor 15 (GDF15), high mobility group box 3 (HMGB3), monocyte to macrophage differentiation-associated (MMD), homeodomain interacting protein kinase 2 (HIPK2) and hypoxia-inducible lipid droplet associated (HILPDA)—are significantly downregulated in TK1 knockdown cells with shRNA. Of these genes, knockdown of both GDF15 and TK1 showed the inability of colonies to form in soft agar in addition to decreased invasion and migration. Additionally, they showed through the knockdown of TK1 that altered levels of GTP/GDP caused deregulation of Rho GTPase activity, leading to a reduction in cancer growth and progression in LUAD cell types [28].

In 2016, Tilli et al. [26] evaluated a siRNA knockdown of network-based targets in MDA-MB-231 cells and the effects on inhibition of tumor development. The network was identified using a systems biology approach and cancer signaling networks to identify five highly expressed and connected proteins: HSP90AB1, CSNK2B, TK1, YWAHB, and VIM. Knockdown of the five proteins was accomplished using siRNA and the transfected cells were assessed in vitro for cell growth, colony formation, migration, and invasion. Colony growth and colony formation were significantly decreased in MDA-MB-231 cells compared to untransfected and scrambled controls. Likewise, migration and invasion were inhibited with siRNA knockdown of target genes in MDA-MB-231 cells when compared to control cell lines MCF7 and MCF-10A. Based on their results, they proposed that this network-based strategy was optimal for inhibiting tumor formation.

Liu et al. [132] were able to isolate individual effects of TK1 on invasion, migration, and cell proliferation in thyroid carcinoma cells. In in vitro experiments, they used normal thyroid cell line Nthy-ori 3-1 and thyroid carcinoma cell lines TPC-1 and BC-PAP. Using siRNA targeting TK1, they were able to determine that knockdown cell lines had decreased ability for invasion, colony formation, and migration. In vivo tumor growth of knockdown TK1 cells lines was analyzed in BALB/nude mice; results reflected those found in in vitro experiments—untreated cells showed significantly more growth in comparison to knockdown TK1 cell lines. Additionally, Western blot analysis showed decreased expression of epithelial-mesenchymal transition (EMT) markers vimentin and N-cadherin. Interestingly, Liu et al. determined that regulatory miRNA miR-34a-5p was downregulated in TPC-1 and BC-PAP cells. When overexpressed through transfected miR-34a-5p mimics there was markedly repressed mRNA and protein expression of TK1 in both cell lines.

TK1 has also been identified bioinformatically among differentially expressed genes (DEGs) as a hub gene involved in adrenocortical carcinoma (ACC) and prostate cancer (PCa) pathogenesis.

Alshabi et al. [25] used extracted microarray datasets GSE19775 from the Gene Expression Omnibus (GEO) database to first identify DEGs before performing subsequent pathway and gene ontology (GO) enrichment analyses. Following this, they did extensive work to identify protein–protein interactions, mutations, miRNA regulatory network and target genes, correlations between hub genes, and prognostic values of hub genes. Eventually, 884 hub genes were identified, with observed upregulation in 441 and downregulation in 443. Pathways that were the most enriched with DEGs included catecholamine biosynthesis, aldosterone synthesis, and secretion, and pyrimidine deoxyribonucleoside salvage. Of the biological processes, the most significant terms were blood vessel morphogenesis and cell cycle phase transition. TK1 was identified as a hub gene related to poor overall survival and as a highly expressed hub gene in stage 4 ACC tumors. This indicates that TK1 may contribute to ACC pathogenesis as well as pathogenesis in other cancer types.

Song et al., performed a similar study in PCa using 10 different extracted microarray datasets from the GEO to identify DEGs associated with PCa pathogenesis [27]. A Robust Rank Aggregation (RRA) method was utilized to compare tumor samples to matched normal samples. From GO and KEGG analyses, the most significant biological process terms were DNA replication and nuclear division. TK1 was among those hub genes that were linked to higher Gleason scores, advanced T grade, and lymph node metastasis in PCa. Additionally, TK1 was within the “DNA replication” set that was highly expressed and involved in PCa tumor proliferation. Interestingly, Song et al. also evaluated tumor purity using TIMER [133]. For TK1 and other hub genes, no association was found between TK1 and infiltration of B cells, CD4^+^ T cells, CD8^+^ T cells, macrophages, neutrophils, or dendritic cells. Lastly, they explored transcriptional regulation of hub genes through methylation data from DiseaseMeth version 2.0 and determined that TK1, LMNB1, RACGAP1, and ZWINT had significantly lower methylation levels in PCa when compared to precancerous samples [133].

## Conclusions

Across many cancer types, serum TK1 has shown effectiveness as a tumor biomarker and, in some cases, to possess advantages in comparison to other biomarkers. It has been determined that increased tissue expression of TK1 is linked to aggressive cancer types and that this upregulation is an early event—as such, TK1 may be used to predict the presence of disease earlier. Multiple sources indicate that TK1 would be beneficial for both prognosis and diagnosis for multiple cancer types when used in cooperation with other clinical tools such as additional biomarkers, laboratory tests, biopsy analyses, and radiological imaging.

Currently, there are immunoassays that have been developed and tested in clinical health screenings that effectively measure serum levels of TK1 [124]. Despite TK1 assay development and clinical setting experimentation, a standardized assay has not been fully adopted to standard of care due to inefficient process times and lack of standard TK1 controls. An improved method that increases the accuracy of TK1 detection in patient serum, as well as decreased processing time, will be necessary if TK1 quantification is to be introduced into clinical practice.

Current studies are now identifying links between TK1 and cancer pathogenesis. Groups such as Tilli et al. [26], Malvi et al. [28], and Liu et al. [132] have shown that TK1 is involved in cancer pathogenesis in vitro in breast, lung, and thyroid cancer respectively. Supporting evidence provided by Song et al. [27] and Alshabi et al. [25] in PCa and ACC shows that TK1 is an enriched gene involved in the progression of both PCa and ACC. Continued research is needed to identify specific networks affected by TK1 in order to elucidate its mechanistic role in cancer pathogenesis. Studying TK1 regulation through in vitro and bioinformatic analyses will be beneficial for identifying the mechanisms behind its dysregulation in cancer and potentially identifying therapeutic targets to inhibit cancer progression.

## Data Availability

Data sharing is not applicable to this article as no datasets were generated or analyzed during the current study.
